# Supplementary material for: The birth of a bacterial tRNA gene by large-scale, tandem duplication events
Source: eLife. 2020 Oct 30;9:e57947. doi: 10.7554/eLife.57947 (PMC7661048; doi:10.7554/eLife.57947)
Supplement: Supplementary file 6. — These include 42 unique primary tRNA sequences, each of which is listed in this file. These 42 sequences are used as references to align the YAMAT-seq data in this work (see Supplementary file 7). Note that the list of 42 sequences includes one likely pseudo tRNA (11_Cys-GCA-2–1). This sequence is not predicted to form a tRNA with conserved cloverleaf secondary structure (Chan and Lowe, 2019). Further, no YAMAT-seq sequences were aligned to this reference sequence in any sample. We conclude that Cys-GCA-2–1 does not form part of the functional mature tRNA pool in SBW25. In addition, the serCGA sequence (30_Ser-CGA-1–1) is expected to be absent from 21 of 27 samples (samples 2–8, 11–17, and 20–26); this tRNA is encoded by serCGA (the gene that was deleted by genetic engineering in this work and remains absent in all derived strains). As expected, almost no reads were obtained for this reference sequence in these 21 samples. The very low numbers of Ser-CGA reads obtained in some of these samples (e.g., two reads in sample 20, the third replicate of ΔserCGA-1) are likely to be barcode misallocations from one of the six SBW25 or W1-L samples. [file elife-57947-supp6.docx]

**Reference list for YAMAT-seq: the 42 unique tRNA sequences in *P. fluorescens* SBW25**

>1_Ala-GGC-1-2 76 bp Sc: 75.2

GGGGCTATAGCTCAGCTGGGAGAGCGCTTGCATGGCATGCAAGAGGtCAACGGTTCGATCCCGTTTAGCTCCACCA

>2_Ala-TGC-1-5 76 bp Sc: 82.7

GGGGCCATAGCTCAGCTGGGAGAGCGCCTGCCTTGCACGCAGGAGGtCAACGGTTCGATCCCGTTTGGCTCCACCA

>3_Arg-ACG-1-2 77 bp Sc: 84.0

GCACTCGTAGCTCAGCTGGAtAGAGTACTCGGCTACGAACCGAGCGGtCACAGGTTCGAATCCTGTCGAGTGCACCA

>4_Arg-CCG-1-1 77 bp Sc: 83.6

GCATCCGTAGCTCAGCTGGAtAGAGTACTGCCCTCCGAAGGCAGGGGtCGTGGGTTCGAATCCCGCCGGGTGCACCA

>5_Arg-CCT-1-1 77 bp Sc: 72.4

GTCCCAGTAGCTCAATTGGAtAGAGCATCCCCCTCCTAAGGGGAAGGtTGGCCGTTCGAACCGGCCCTGGGACACCA

>6_Arg-TCT-1-1 77 bp Sc: 89.3

GCGCCCGTAGCTCAGCTGGAtAGAGCATCCGCCTTCTAAGCGGATGGtCGCAGGTTCGAGTCCTGCCGGGTGCGCCA

>7_Asn-GTT-1-1 76 bp Sc: 80.6

TCCGTGATAGCTCAGTCGGTAGAGCAAATGACTGTTAATCATTGGGtCCCAGGTTCGAGTCCTGGTCACGGAGCCA

>8_Asn-GTT-2-1 76 bp Sc: 77.6

TCCGCGATAGCTCAGTTGGTAGAGCAAATGACTGTTAATCATTGGGtCCCTGGTTCGAGTCCAGGTCGTGGAGCCA

>9_Asp-GTC-1-4 77 bp Sc: 90.7

GCAGCGGTAGTTCAGTCGGTtAGAATACCGGCCTGTCACGCCGGGGGtCGCGGGTTCGAGTCCCGTCCGCTGCGCCA

>10_Cys-GCA-1-1 74 bp Sc: 65.3

GGCCGAGTAGCAAAATGGTTATGCAGCGGATTGCAAATCCGCCTaCGCCGGTTCGATTCCGACCTCGGCCTCCA

>11_Cys-GCA-2-1 112 bp Sc: 23.4 (pseudo tRNA, no reads align in any sample)

GAGTAAATGTTGGTgAtgcggaatagattatcatataacttattgaaaataatgaggaTATTCGTGGATTGCAAATCCGCCTaCGCCGGTTCGATTCCGACCTCGGCCTCCA

>12_Gln-TTG-1-1 75 bp Sc: 69.7

AGGGGCGTCGCCAAGCGGTAAGGCAGCAGGTTTTGATCCTGCCATgCGTTGGTTCGAATCCAGCCGCCCCTGCCA

>13_Glu-TTC-1-4 76 bp Sc: 68.9

GTCCCCTTCGTCTAGTGGCctAGGACACCGCCCTTTCACGGCGGTAaCAGGGGTTCGAGTCCCCTAGGGGACGCCA

>14_Gly-CCC-1-1 74 bp Sc: 74.9

GCGGGTATAGTTTAATGGTAGAACAGTAGCTTCCCAAGCTTCCGaCGAGGGTTCGATTCCCTCTACCCGCTCCA

>15_Gly-GCC-1-3 76 bp Sc: 88.4

GCGGGAATAGCTCAGTTGGTAGAGCACGACCTTGCCAAGGTCGGGGtCGCGAGTTCGAGTCTCGTTTCCCGCTCCA

>16_Gly-TCC-1-1 74 bp Sc: 83.2

GCGGGTATAGTTTAGTGGTAGAACCTCAGCCTTCCAAGCTGATGaTGCGGGTTCGATTCCCGCTACCCGCTCCA

>17_His-GTG-1-2 76 bp Sc: 73.3

GTGGGCGTAGCTCAGTTGGTAGAGCACGGGATTGTGACTCCCGTTGtCGAGGGTTCGATCCCCTTCGTCCACCCCA

>18_Ile-GAT-1-5 77 bp Sc: 88.9

GGGTCTGTAGCTCAGTTGGTtAGAGCGCACCCCTGATAAGGGTGAGGtCGGCAGTTCGAATCTGCCCAGACCCACCA

>19_Ile2-CAT-1-1 77 bp Sc: 89.4

GGGCCTATAGCTCAGTTGGTtAGAGCAGGGGACTCATAATCCCTTGGtCGTAGGTTCGAGTCCTACTGGGCCCACCA

>20_Leu-CAA-1-1 85 bp Sc: 70.8

GCCCTGATGGCGGAATTGGTaGACGCGGCGGATTCAAAATCCGTTTTCGAAAgGAGTGGGAGTTCGAGTCTCCCTCGGGGCACCA

>21_Leu-CAG-1-2 87 bp Sc: 71.6

GCCCAGGTGGTGAAATTGGTaGACACGCCAGCTTCAGGTGCTGGTGATCGCAAGGTCGTGGAAGTTCGAGTCTTCTCCTGGGCACCA

>22_Leu-GAG-1-1 86 bp Sc: 60.3

GCCGAGGTGGTGGAATTGGTaGACACGCAACCTTGAGGTGGTTGTGCCCATAGGGTgTAGGGGTTCGAGTCCCCTTCTCGGTACCA

>23_Leu-TAA-1-1 87 bp Sc: 71.0

GCCCGAATGGCGAAACTGGTaGACGCATGGGACTTAAAATCCCCCGCTCGTAAGGGCGTCCCGGTTCGATTCCGGGTTCGGGCACCA

>24_Leu-TAG-1-1 85 bp Sc: 70.1

GCGGATGTGGTGGAATTGGTaGACACACTGGATTTAGGTTCCAGCGCCGCGAGGCGTAAGAGTTCGAGTCTCTTCATCCGCACCA

>25_Lys-TTT-1-2 76 bp Sc: 86.5

GGGTCGTTAGCTCAGTTGGTAGAGCAGTTGGCTTTTAACCAATTGGtCGTAGGTTCGAATCCCACACGACCCACCA

>26_Met-CAT-1-1 77 bp Sc: 77.5

GGCTACATAGCTCAGTTGGTtAGAGCATAGCATTCATAATGCTGGGGtCCGGGGTTCAAGTCCCTGTGTAGCCACCA

>27_Phe-GAA-1-1 76 bp Sc: 81.1

GCCCAGATAGCTCAGTCGGTAGAGCAGGGGATTGAAAATCCCCGTGtCGGCGGTTCGATTCCGTCTCTGGGCACCA

>28_Pro-GGG-1-1 77 bp Sc: 67.6

CGGGGCGTAGCGCAGTCcGGTAGCGCACTAGCATGGGGTGCTAGGGGtCGAGTGTTCGAATCACTCCGTCCCGACCA

>29_Pro-TGG-1-2 77 bp Sc: 73.4

CGGGGTATAGCGCAGTCcGGTAGCGCGCCTGCTTTGGGAGCAGGATGtCAGGAGTTCGAATCCCCTTACCCCGACCA

>30_Ser-CGA-1-1 90 bp Sc: 71.0

GGAGAGATGCCAGAGTGGCcgAATGGGACGGATTCGAAATCCGTTGTACCTTCACCGGTACCTAGGGTTCGAATCCCTATCTCTCCGCCA

>31_Ser-GCT-1-1 91 bp Sc: 75.0

GGAGAGCTGGCCGAGTGGCcgAAGGCGCTCCCCTGCTAAGGGAGTACACCTCAAaAGGGTGTCGGGGGTTCGAATCCCCCGTTCTCCGCCA

>32_Ser-GGA-1-1 90 bp Sc: 75.7

GGTGAAGTGTCCGAGTGGCttAAGGAGCACGCCTGGAAAGTGTGTATACAAGAAATTGTATCGAGAGTTCGAATCTCTCCTTCACCGCCA

>33_Ser-TGA-1-1 91 bp Sc: 75.4

GGGAAATTGGCAGAGTGGTtgAATGCACCGGTCTTGAAAACCGGCGGACGTTAAtAGCGTCTCCAGGGTTCGAATCCCTGGTTTCCCGCCA

>34_Thr-CGT-1-1 73 bp Sc: 69.7

GCCCGTGTAGCTCAGTCGGTAGAGCAGCGCACTCGTAACGCGAAGGtCGCAGGTTCGATTCCTGTCTCGGGCACCA

>35_Thr-GGT-1-1 76 bp Sc: 80.7

GCTCTTGTAGCTCAGTTGGTAGAGCACACCCTTGGTAAGGGTGAGGtCAGCGGTTCAAATCCGCTCAAGAGCTCCA

>36_Thr-TGT-1-1 76 bp Sc: 87.9

GCCGGTATAGCTCAGTTGGTAGAGCAACTGACTTGTAATCAGTAGGtCCCGGGTTCGACTCCTGGTGCCGGCACCA

>37_Trp-CCA-1-1 76 bp Sc: 86.5

AGGTCAGTAGCTCAATTGGCAGAGCGACGGTCTCCAAAACCGTAGGtTGGGGGTTCGATTCCCTCCTGACCTGCCA

>38_Tyr-GTA-1-1 85 bp Sc: 77.1

GGAGGGGTTCCCGAGCGGCcaAAGGGATCAGACTGTAAATCTGACGTCTACGACTtCGAAGGTTCGAATCCTTCCCCCTCCACCA

>39_Val-GAC-1-1 77 bp Sc: 78.1

AGGCACGTAGCTCAGTTGGTtAGAGCACCACCTTGACATGGTGGGGGtCGTTGGTTCGAGTCCAATCGCGCCTACCA

>40_Val-TAC-1-3 76 bp Sc: 84.4

GGGTGATTAGCTCAGCTGGGAGAGCATCTGCCTTACAAGCAGAGGGtCGGCGGTTCGATCCCGTCATCACCCACCA

>41_fMet-CAT-1-1 77 bp Sc: 78.4

CGCGGGGTGGAGCAGTCtGGTAGCTCGTCGGGCTCATAACCCGAAGGtCGTCGGTTCAAATCCGGCCCCCGCAACCA

>42_fMet-CAT-2-2 77 bp Sc: 76.7

CGCGGGATGGAGCAGTCtGGTAGCTCGTCGGGCTCATAACCCGAAGGtCGTCGGTTCAAATCCGGCTCCCGCAACCA
