# Supplementary material for: The birth of a bacterial tRNA gene by large-scale, tandem duplication events
Source: eLife. 2020 Oct 30;9:e57947. doi: 10.7554/eLife.57947 (PMC7661048; doi:10.7554/eLife.57947)
Supplement: Transparent reporting form [file elife-57947-transrepform.pdf]

eLIFE
1st Floor
24 Hills Road
Cambridge CB2 1JP, UK
P 01223 855340
W elifesciences.org
T @elife

eLife's transparent reporting form

We encourage authors to provide detailed information *within their submission* to facilitate the interpretation and replication of experiments. Authors can upload supporting documentation to indicate the use of appropriate reporting guidelines for health-related research (see EQUATOR Network), life science research (see the BioSharing Information Resource), or the ARRIVE guidelines for reporting work involving animal research. Where applicable, authors should refer to any relevant reporting standards documents in this form.

If you have any questions, please consult our Journal Policies and/or contact us: editorial@elifesciences.org.

Sample-size estimation

- You should state whether an appropriate sample size was computed when the study was being designed
- You should state the statistical method of sample size computation and any required assumptions
- If no explicit power analysis was used, you should describe how you decided what sample (replicate) size (number) to use

Please outline where this information can be found within the submission (e.g., sections or figure legends), or explain why this information doesn't apply to your submission:

This information is contained within the following Materials and Methods sections: "Growth curves" (and the legends of Figures 2, 3, 4), "Fitness assays" (and the legends of Figures 2, 3), "Evolution experiment", "Expression of tRNA genes from the pSXn plasmid" (and the legend of Figure 4), and "YAMAT-seq" (and the legends of Figures 5, and Figure 5 – supplemental figure 1).

Replicates

- You should report how often each experiment was performed
- You should include a definition of biological versus technical replication
- The data obtained should be provided and sufficient information should be provided to indicate the number of independent biological and/or technical replicates
- If you encountered any outliers, you should describe how these were handled
- Criteria for exclusion/inclusion of data should be clearly stated
- High-throughput sequence data should be uploaded before submission, with a private link for reviewers provided (these are available from both GEO and ArrayExpress)

Please outline where this information can be found within the submission (e.g., sections or figure legends), or explain why this information doesn't apply to your submission:

eLife Sciences Publications, Ltd is a limited liability non-profit non-stock corporation incorporated in the State of Delaware, USA, with company number 5030732, and is registered in the UK with company number FC030576 and branch number BR015634 at the address 1st Floor, 24 Hills Road, Cambridge CB2 1JP | August 2014

Information on numbers of replicates is detailed within the legends of Figures 2, 3, 4, 5, and Figure 5 – supplemental figure 1. Details of replication methods are found in Materials and Methods sections "Strains and growth conditions", "Growth curves", "Fitness assays", "Evolution experiment", "Expression of pSX genes from the pSXn plasmid", and "YAMAT-seq".

Information on the exclusion of data from analyses (one growth curve replicate from Figures 2B and 2C; plus three tRNA isotypes from the DESeq2 analysis) can be found in source data file 1, and the results section "Duplication events increase the proportion of tRNA-Ser(UGA) in the mature tRNA pool", respectively.

Illumina whole genome sequencing data has been uploaded to NCBI SRA (accession PRJNA558233).

YAMAT-seq data has been uploaded to NCBI GEO (accession GSE144791).

Statistical reporting

- Statistical analysis methods should be described and justified
- Raw data should be presented in figures whenever informative to do so (typically when N per group is less than 10)
- For each experiment, you should identify the statistical tests used, exact values of N, definitions of center, methods of multiple test correction, and dispersion and precision measures (e.g., mean, median, SD, SEM, confidence intervals; and, for the major substantive results, a measure of effect size (e.g., Pearson's r, Cohen's d)
- Report exact p-values wherever possible alongside the summary statistics and 95% confidence intervals. These should be reported for all key questions and not only when the p-value is less than 0.05.

Please outline where this information can be found within the submission (e.g., sections or figure legends), or explain why this information doesn't apply to your submission:

Statistical methods are described in the Materials and Methods sections "Analysis of YAMAT-seq data", and "Statistical tests".

Further information, including tests used, values of N, and measures of centre are provided in the legends of Figures 2, 3, 4, 5, and Figure 5 – supplemental figure 1, plus source data sets 1-5.

(For large datasets, or papers with a very large number of statistical tests, you may upload a single table file with tests, Ns, etc., with reference to sections in the manuscript.)

Group allocation

- Indicate how samples were allocated into experimental groups (in the case of clinical studies, please specify allocation to treatment method); if randomization was used, please also state if restricted randomization was applied
- Indicate if masking was used during group allocation, data collection and/or data analysis

Please outline where this information can be found within the submission (e.g., sections or figure legends), or explain why this information doesn't apply to your submission:

There is no group allocation in this manuscript.

Additional data files ("source data")

- We encourage you to upload relevant additional data files, such as numerical data that are represented as a graph in a figure, or as a summary table
- Where provided, these should be in the most useful format, and they can be uploaded as "Source data" files linked to a main figure or table
- Include model definition files including the full list of parameters used
- Include code used for data analysis (e.g., R, MatLab)
- Avoid stating that data files are "available upon request"

Please indicate the figures or tables for which source data files have been provided:

eLIFE
1st Floor
24 Hills Road
Cambridge CB2 1JP, UK
P 01223 855340
W elifesciences.org
T @elife

Figure 2 – source data 1 pertains to Figures 2B, 2C, 2F, and 2G.
Figure 2 and 3 – source data 2 pertains to Figures 2D, 2H, and 3E.
Figure 3 – source data 3 pertains to Figures 3B, 3C, and 3D.
Figure 4 – source data 4 pertains to Figures 4C, and 4D.
Figure 5 – source data 5 pertains to Figures 5A, 5B, Figure 5 supplemental figure 1.

Figure 1 – supplemental figure 1: Predicted structure and function of tRNA types tRNA-Ser(CGA) and tRNA-Ser(UGA) in *P. fluorescens* SBW25.
Figure 2 – supplemental figure 1: The effect of *serCGA* deletion on cell morphology during growth in liquid KB and M9.
Figure 4 – supplemental figure 1: Coverage plots from whole genome sequencing data provide evidence of large-scale, tandem duplication events in evolutionary lineages M1-M4.
Figure 4 – supplemental figure 2: Large tandem duplication are first detected between Days 2 and 5 of the evolution experiment.
Figure 5 – supplemental figure 1: Comparison of expression levels of tRNA types in five strains isolated from mutant lineages on Day 13.

Supplemental file 1: excel file containing details of the tRNA types predicted in *P. fluorescens* SBW25, and predicted codon-tRNA matching patterns.
Supplemental file 2: excel file containing details of strains, plasmids, oligonucleotides, duplication junctions used in this study.
Supplemental file 3: excel file detailing the results of Illumina whole genome sequencing, including raw read numbers and predicted mutations.
Supplemental file 4: excel file detailing the genes duplicated in each of the five Day 13 isolates from mutant lineages of the evolution experiment.
Supplemental file 5: excel file containing information on base variations in the NGS sequence alignment across the *serTGA* gene and promoter, in the Day 13 evolution experiment isolates.
Supplemental file 6: word document listing the reference tRNA sequences used for alignment of YAMAT-seq data (*i.e.*, the 42 unique primary tRNA gene structures predicted in SBW25).
Supplemental file 7: excel file detailing the YAMAT-seq results, including numbers of raw and processed reads, and read numbers aligned to reference tRNAs (see supplemental file 6) in each strain.
